# Reply to: On the existence of collective interactions reinforcing the metal-ligand bond in organometallic compounds

**DOI:** 10.1038/s41467-023-39504-3

**Published:** 2023-07-03

**Authors:** Vojtech Šadek, Shahin Sowlati-Hashjin, SeyedAbdolreza Sadjadi, Mikko Karttunen, Angel Martín-Pendás, Cina Foroutan-Nejad

**Affiliations:** 1 Department of Chemistry, Faculty of Science, Masaryk University, Kamenice 5, CZ-62500 Brno, Czechia; 2 CEITEC–Central European Institute of Technology, Masaryk University, Kamenice 5, CZ-62500 Brno, Czechia; 3 Institute of Biomedical Engineering, University of Toronto, Toronto, ON M5S 3G9 Canada; 4 Faculty of Science, Laboratory for Space Research, The University of Hong Kong, Hong Kong SAR, China; 5 Department of Chemistry, The University of Western Ontario, 1151 Richmond Street, N6A 3K7 London, Ontario Canada; 6 Department of Physics and Astronomy, The University of Western Ontario, 1151 Richmond Street, London, ON N6A 5B7 Canada; 7 Centre for Advanced Materials and Biomaterials Research, The University of Western Ontario, 1151 Richmond Street, London, ON N6K 3K7 Canada; 8 Departamento de Química Física y Analítica, University of Oviedo, 33006 Oviedo, Spain; 9 Institute of Organic Chemistry, Polish Academy of Sciences, Kasprzaka 44/52, 01-224 Warsaw, Poland

**Keywords:** Computational chemistry, Chemical bonding

replying to J. Poater et al. *Nature Communications* 10.1038/s41467-023-39498-y (2023)

Poater et al. (PVHBS) questioned our proposal for a new type of bond, collective interactions, CI^1^, between certain species with a general formula M^n+^AX_3_^m‒^ using an energy decomposition analysis, EDA^2,3^. Here, we show that their work confirms the existence of collective bonding. Before going further we should emphasize that unlike our methodology, the theory of interacting quantum atoms (IQA)^4^, conventional EDA analyses cannot assess the nature of a given interatomic interactions, e.g., M…A, within M^n+^AX_3_^m‒^ complexes simply because atoms or other subgroups in a fragment are not defined in EDA. EDA can only provide a picture of the inter-fragment interactions between the M and the atoms in AX_3_ fragments.

PVHBS start their argument by assuming that LiCF_3_ and LiC(Ph)_3_ both have covalent bonds between Li and the neighboring C atoms. Comparing the relative contributions of exchange-correlation, which we call the covalent component from now on, and the ionic interactions between atoms or fragments, Table 1 and Fig. 1 in the original paper, show that Li…C interaction in LiCF3 is dominantly ionic. The local interaction between Li and C is composed of a repulsive electrostatic component (93.8 kcal/mol) and a slightly attractive covalent part (−21.3 kcal/mol) therefore, the local interaction of Li and C in LiCF_3_ is repulsive. However, the overall stabilization stems from the ionic interaction between the Li^+^ cation and CF_3_^‒^ anion in which the negative charge is concentrated on the F atoms. Thus, the inter-fragment interaction of Li^+^ with CF_3_^‒^ is dominantly an ionic one. This is consistent with the widely accepted (though recently questioned^5,6^) proposal in the EDA community that the fragmentation scheme showing the smallest |ΔE_oi_| provides the best description of the electronic state of the interacting atoms^7–10^; see PVHBS data in their Supplementary Table 1 (ΔE_oi_ = −95.5 versus 19.9 for homolytic and heterolytic dissociation). The interatomic interaction between Li^+^ and the C *atom* in CF_3_^‒^ is; however, a unique interaction between a metal and a nonmetal that has characteristics of covalent interaction because its ionic component is repulsive (destabilizing) but its covalent (exchange-correlation) contribution is attractive (stabilizing). On the other hand, Li…C interaction in LiC(Ph)_3_ has both attractive electrostatic (−49.6 kcal/mol) and covalent (−10.7 kcal/mol) components simply because Ph is not as electronegative as F atoms and more negative charge can rest on C. While the covalent interaction between each Li…F is only −0.7 kcal/mol (that is 0.33% of Li…C covalent component), the Li…Ph covalent component is 5.5 kcal/mol, that is, 51.40% of Li…C interaction in the molecule. In other words, in LiC(Ph)_3_ the covalent interaction is collective between all parts of the molecule. PVHBS explain the weaker Li…C interaction in LiC(Ph)_3_ by Pauli repulsion. IQA analysis suggests that the source of the lower Li…C interaction energy is simply weaker electrostatics. On the other hand, IQA justifies the fact that in LiC(Ph)_3_ the C(Ph)_3_ structure is inverted, i.e., the C‒C‒Li angle is 83.27°. If Pauli repulsion is a real effect, we would have expect to find a pyramidal LiC(Ph)_3_ that was never found on the gas-phase PES of the molecule.

**Fig. 1:**
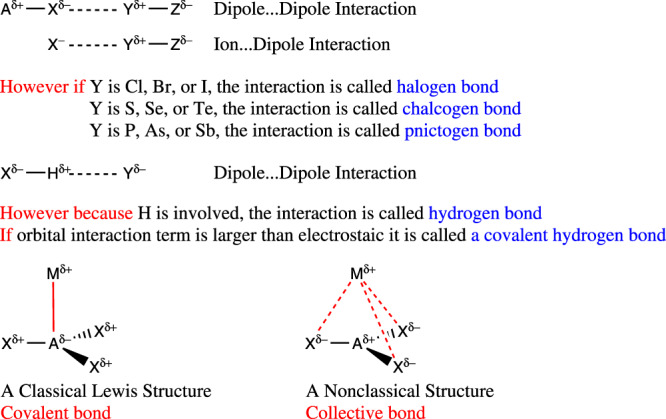
Collective bonds deserve recognition. While one can classify nearly all halogen, chalcogen, and pnictogen, even hydrogen bonds among either dipole…dipole or ion…dipole interactions, the community now acknowledges the different names because that helps to recognize them better once, we see them in a structure. A nonclassical collective bond (interaction) deserves recognition because the mechanism of collective bond formation is essentially different from classical covalent, dative, or polar-covalent bonds.

IQA^4^, clearly shows that the origin of CI is the quantum mechanical exchange-correlation energy that lies at the root of covalent interactions. Thus, CI is a covalent-like interaction between the M and X atoms, which are not related to a bonded Lewis structure. Of course, and as expected, a non-negligible ionic interaction between the positively charged M and the negatively charged Xs in the MAX_3_ moieties strengthens CI in many structures.

PVHBS continue that, according to their analysis, 1,3 interactions in LiCPh_3_ reduce the Li‒C overlap and conclude that this interaction should be destabilizing. PVHBS’ Supplementary Table 1 shows that in their model systems, LiCPh_3_, LiCPh_2_^•^, and LiCPh^••^, the Li–C overlap changes from 0.071, which is barely bonding, to 0.167, and 0.229, respectively. Irrespective of the physical/chemical validity of these unoptimized DFT-based models that fail a T-test^11^, Supplementary Table S1, PVHBS’ Supplementary Table 1 shows that while the overlap integral changes by more than 300%, the orbital interaction energy remains essentially the same for LiCPh_3_ and LiCPh^••^ (−98.2 and −98.5 kcal/mol), and surprisingly drops for LiCPh_2_^•^ to only −83.2 kcal/mol when just one phenyl is removed. This clearly shows that while the overlap between Li and the central C atom is strengthened in this series, a stabilization factor is lost and the tradeoff between the lost factor and the extra gained overlap in the process keeps the ΔE_oi_ values constant. On the contrary, for classical LiCF_3_ the removal of the F atoms has a negligible effect on both the overlap and, thus, the ΔE_oi_ values, as expected. We propose that the stabilization, which is proportional to the number of phenyl groups, comes from the collective interaction. Because the ΔE_oi_ values for LiCPh(F)_3_ series do not change, if PVHBS would like to extract their own *ICI*_*XC*_*-like* index they could divide the overlap for the LiCPh(F)_3_ system by that of LiCPh(F)^••^ to obtain *ICI*_*XC*_*-EDA*(LiCPh_3_) = 0.310 and *ICI*_*XC*_*-EDA*(LiCF_3_) = 0.961, close to our original values (ICI_XC_(LiCPh_3_) = 0.393 and ICI_XC_(LiCF_3_) = 0.910). Note that we are not advocating for a general recipe to compute ICI_XC_ values based on EDA; in this case the identical ΔE_oi_ values permit an evaluation of an ICI_XC_-like index with EDA descriptors.

We should stress that the variation of the magnitude of the EDA Pauli repulsion component, as discussed before, is not a reliable tool to assess the nature of a bond because ΔE_Pauli_ can be tuned at will if one uses a different EDA scheme, e.g., a step-wise EDA^12,13^. Nevertheless, it is interesting to note that, according to PVHBS’ Supplementary Table 1, in the heterolytic fragmentation of LiCPh_3_ to a carbanion and Li^+^, which should be taken as the most realistic bonding model based on its smaller |ΔE_oi_| within the EDA paradigm^7–10^, the ΔE_Pauli_ repulsion between the phenyl rings and Li^+^ does not exist because it barely decreases from 18.9 kcal/mol in LiCPh3 to 17.1 kcal/mol for LiCPh_2_^•^ and again increases to 19.9 kcal/mol in LiCPh^••^. It is curious to note that ΔE_Pauli_ for pyramidal LiCF_3_, a species that according to PVHBS should not have steric strain between its F substituents and the Li atom, decreases in the heterolytic dissociation channel according to their computations (ΔE_Pauli_ = 34.9, 28.0, and 25.1 kcal/mol for LiCF_3_, LiCF_2_^•^, and LiCF^••^, respectively)!

Inspection of the EDA data listed in PVHBS’ Supplementary Tables 1 and 2 for *i*-LiCF_3_ and LiCF_3_, two closely related species with and without collective interaction, is illuminating and avoids any unoptimized radical model system. PVHBS write that collective interaction weakens bonds because of Pauli repulsion even in inverted *i*-LiCF_3_, as compared to the pyramidal form of the molecule, LiCF_3_. However, they fail to mention that (1) *i*-LiCF_3_ is the global minimum on the potential energy surface of the molecule, (2) the Li‒C bond dissociation energy according to their computations is −66.0 kcal/mol for the homolytic and −152.4 kcal/mol for the heterolytic dissociation of Li in *i*-LiCF_3_ versus −63.6 and −150.0 kcal/mol for the homolytic and heterolytic dissociation of the pyramidal LiCF_3_. This is so despite a much larger Pauli component in *i*-LiCF_3_ (128.7 kcal/mol homolytic, 21.0 kcal/mol heterolytic) versus pyramidal LiCF_3_ (46.6 kcal/mol homolytic, 34.9 kcal/mol heterolytic), even if we neglect the fact that the Pauli term for heterolytic dissociation of *i*-LiCF_3_ is notably smaller than the corresponding value for the pyramidal isomer.

Another interesting point that is overlooked by PVHBS is that despite a much smaller overlap in *i*-LiCF_3_ (0.219) as compared to LiCF_3_ (0.317), the orbital interaction energy of *i*-LiCF_3_ is significantly higher (−155.4 kcal/mol) than in the pyramidal isomer (−95.5 kcal/mol) according to data in their Supplementary Tables 1 and 2. If we accept PVHBS’ proposal that the out-of-phase overlap is stronger in *i*-LiCF_3_, then either the orbital interaction term is not appropriately showing the orbital interaction energies, or the analysis is missing an essential part which is the collective interaction.

Finally, as PVHBS conclude, and as we mentioned earlier, collective bonding arises when the stabilizing interaction between two immediate neighboring atoms is negligible and, either because of the distance or unfavorable charges like in LiCF_3_ or NaBH_3_^‒^^14^, there is a repulsive (destabilizing) ionic interaction between the M and A atoms in the MAX_3_ molecules, but the M and X interaction is attractive (stabilizing). In that sense, collective bonds have not been identified before and perhaps could not be identified without using an appropriate partitioning method (Fig. 1). While in some species with collective bonds electrostatic component plays a more notable role in bonding than the covalent part, it is not always the case and no matter which factor is dominant, ICI_XC_ is a sensitive probe to identify nonclassical interactions.

## Supplementary information

Supplementary Information

## Data Availability

The results of the *T*-test, performed on intermediates, are shown in the Supplementary Information.
