# Supplementary material for: Reply to: On the existence of collective interactions reinforcing the metal-ligand bond in organometallic compounds
Source: Nat Commun. 2023 Jul 3;14:3873. doi: 10.1038/s41467-023-39504-3 (PMC10317954; doi:10.1038/s41467-023-39504-3)
Supplement: Supplementary file 1 — Supplementary Information [file 41467_2023_39504_MOESM1_ESM.pdf]

Supplementary Information

Reply to: On the existence of collective interactions

Vojtech Šadek[1,2], Shahin Sowlati-Hashjin[3], SeyedAbdolreza Sadjadi[4], Mikko Karttunen[5,6,7], Angel Martín-Pendás[8*] & Cina Foroutan-Nejad[9*]

1. Department of Chemistry, Faculty of Science, Masaryk University, Kamenice 5, CZ-62500 Brno, Czechia.
2. CEITEC − Central European Institute of Technology, Masaryk University, Kamenice 5, CZ-62500 Brno, Czechia.
3. Institute of Biomedical Engineering, University of Toronto, Toronto, ON M5S 3G9, Canada.
4. Department of Physics, Faculty of Science, Laboratory for Space Research, The University of Hong Kong, Hong Kong SAR, China.
5. Department of Chemistry, The University of Western Ontario, 1151 Richmond Street, London, Ontario, Canada N6A 3K7.
6. Department of Physics and Astronomy, The University of Western Ontario, 1151 Richmond Street, London, Ontario N6A 5B7, Canada.
7. Centre for Advanced Materials and Biomaterials Research, The University of Western Ontario, 1151 Richmond Street, London, Ontario, Canada N6K 3K7.
8. Departamento de Química Física y Analítica, University of Oviedo, 33006 Oviedo, Spain.
9. Institute of Organic Chemistry, Polish Academy of Sciences, Kasprzaka 44/52, 01-224 Warsaw, Poland.

Corresponding authors' emails:
AMP: ampendas@uniovi.es
CFN: cina.foroutan-nejad@icho.edu.pl

Supplementary Table 1. T-test for radical and diradical model systems of PVHBS. The values are computed at CCSD/def2-SVP level. The values larger than 0.02 suggest that neither DFT nor any single-reference method are not a reliable approach to assess the wavefunction because the systems have multireference character, as it has been discussed elsewhere.[11]

Molecules	T-value	Molecules	T-value	Molecules	T-value
$LiCPh_2^{\bullet}$	0.040	$LiCF_2^{\bullet}$	0.019	i-$LiCF_2^{\bullet}$	0.017
$LiCPh^{\bullet\bullet}$	0.038	$LiCF^{\bullet\bullet}$	0.024	i-$LiCF^{\bullet\bullet}$	0.021
